# Cover crops and chicken grazing in a winter fallow field improve soil carbon and nitrogen contents and decrease methane emissions

**DOI:** 10.1038/s41598-020-69407-y

**Published:** 2020-07-28

**Authors:** Huabin Zheng, Linhong Zhou, Jiabing Wei, Qiyuan Tang, Yingbin Zou, Jianwu Tang, Huaqin Xu

**Affiliations:** 1 College of Agronomy, Hunan Agricultural University, Changsha, 410128 China; 2 The Ecosystems Center, Marine Biological Laboratory, Woods Hole, MA 02543 USA

**Keywords:** Field trials, Ecosystem services

## Abstract

Using symbiotic farming methods [cover crops and chicken grazing (+ C)] in a winter fallow field, we found that the soil organic matter and total nitrogen of the + C treatment were 5.2% and 26.6% higher, respectively, than those of a treatment with cover crops and no chicken grazing (− C). The annual rice grain yield of the + C treatment was 3.8% higher than that of the − C treatment and 12.3% higher than that of the bare fallow field (CK), while the annual CH_4_ emissions of the + C treatment were 26.9% lower than those of the − C treatment and 10.6% lower than those of the CK treatment. The 100-year global warming potential of the + C treatment was 6.2% lower than that of the − C treatment. Therefore, the use of winter cover crops and chicken grazing in a winter fallow field was effective at reducing CH_4_ emissions and significantly improving soil nutrients and rice yield.

## Introduction

South China is a main double-season rice production area^1^. Intensification of rice cultivation may pose risks to the long-term sustainability and productivity of rice paddies^2^. At present, the development of paddy field farming systems is faced with outstanding problems and challenges such as reduction of ripening system, excessive input, waste of resources, weakening of cultivated land, fragile foundation and reduced efficiency^3,4^. Therefore, there is an urgent need to develop a soil management practice that will improve soil quality and eliminate the risks associated with intensified double rice cropping in southern China.

In south China, the paddy field has a large winter leisure area, most areas have a suitable climate and abundant resources. However, the multiple cropping index is low, which is a great waste of natural resources and reduces soil fertility. Green manuring is considered as an important soil management practice with the potential to maintain soil organic matter (SOM) content and reduce the dependence on mineral fertilizers^5^. The crops used in green manuring are generally high biomass yielding and N-fixing plant species^6,7^. In addition, the growing period of the cover crop does not lead to competition over land for rice production. The growth duration of the double rice cropping season after transplantation lasts from April to October^8^, while November to March (the winter season) of the following year can be used to plant green manure in most regions in southern China. Green manure returning to the field has improved soil physical and chemical and biological properties, increased crop yield, and controlled paddy field’s weed and other functions. The cultivation of winter cover crops as green manure has also been strongly recommended in rice paddy soil in temperate-zone countries, such as Korea and Japan^9^. Similarly, in southern China, farmers often seed cover crops after rice harvest and then incorporate them in situ as green manure before transplanting rice in the next season. However, the low economic benefits of cover cropping often prevent the large deployment of this practice. Furthermore, the addition of cover crops as green manure could stimulate CH_4_ emissions in flooded paddy soil^10–13^. Therefore, additional studies are needed to improve soil fertility, including SOM and liable nitrogen, while reducing CH_4_ emissions for cover cropping management.

Symbiotic farming technologies, such as rice-fish farming and rice-duck farming, could significantly reduce greenhouse gas emissions (as reviewed by Zheng et al.^14^) in addition to the benefits that the symbiotic systems have on rice paddies^14,15^. In this study, it was found that the cover crops (high biomass yield and protein) used for chicken grazing could reduce the cost of feeding the chickens. Any remaining grass is eaten by the chickens, and the manure produced further enhances the soil. Green manure straw is good chicken feed as it has high protein. With moderate chicken intensity, the green manure continued to grow after the chicken were sold. And finial the mixture with chicken manure and straw enhances the soil. This system of cover crop planting and chicken grazing was relatively simple to maintain, reducing labor costs. The free-range chickens are then sold before the main crops are planted, thus increasing the economic outputs. In this study, using a symbiotic farming practice with cover crops with chicken grazing in a winter fallow field, we aim to (i) improve soil carbon and nitrogen availability, (ii) increase rice grain yield, and (iii) reduce CH_4_ emissions in the rice growing season.

## Results

### Soil organic carbon and nitrogen

The soil organic carbon content in the plot with cover crops was significantly higher than that in the bare fallow field (CK, Table 1). The soil organic carbon in the cover crop and chicken grazing (+ C) treatment was 5.2% (3 ~ 8%) higher than that in the treatment with cover crops and no chicken grazing (− C). Similarly, the soil total nitrogen content in the plot with cover crops was significantly higher than that in the CK plot (Table 1). The soil total nitrogen in the + C treatment was 26.6% (18 ~ 41%) higher than that in the − C treatment. The C:N ratio of the CK was significantly higher than that of the plot with cover crops, and the C:N ratio in the + C treatment was 16.5% (11 ~ 23%) lower than that in the − C treatment, while no significant difference was observed between the + C and − C treatments in the rice planting seasons. Therefore, chicken grazing in the cover crop plots increased the soil organic carbon and total nitrogen.

**Table 1:** Variation in the soil organic carbon and total nitrogen among the three treatments [cover crops with chicken grazing (+ C), cover crops with no chicken grazing (− C), and bare fallow field (CK)].

Treatment	Soil organic carbon (g kg^−1^)			Soil total nitrogen (g kg^−1^)			C:N ratio		
	GM	ER	LR	GM	ER	LR	GM	ER	LR
+ C	15.20a	14.32a	14.74a	3.24a	2.80a	2.50a	4.69c	5.11b	5.88b
− C	14.10a	13.63a	14.34a	2.30b	2.38a	2.06b	6.12b	5.72b	6.97b
CK	12.41b	11.91b	12.53b	1.05c	1.36b	1.18c	11.85a	8.79a	10.61a

GM, ER, and LR were the green manure return, early rice harvest and late rice harvest stages, respectively; the C:N ratio was the ratio between the soil organic matter and soil total nitrogen. Different lowercase letters in the same column among the different treatments indicate significant differences at the 0.05 level.

### Soil microbial carbon and nitrogen

**Figure 1:**
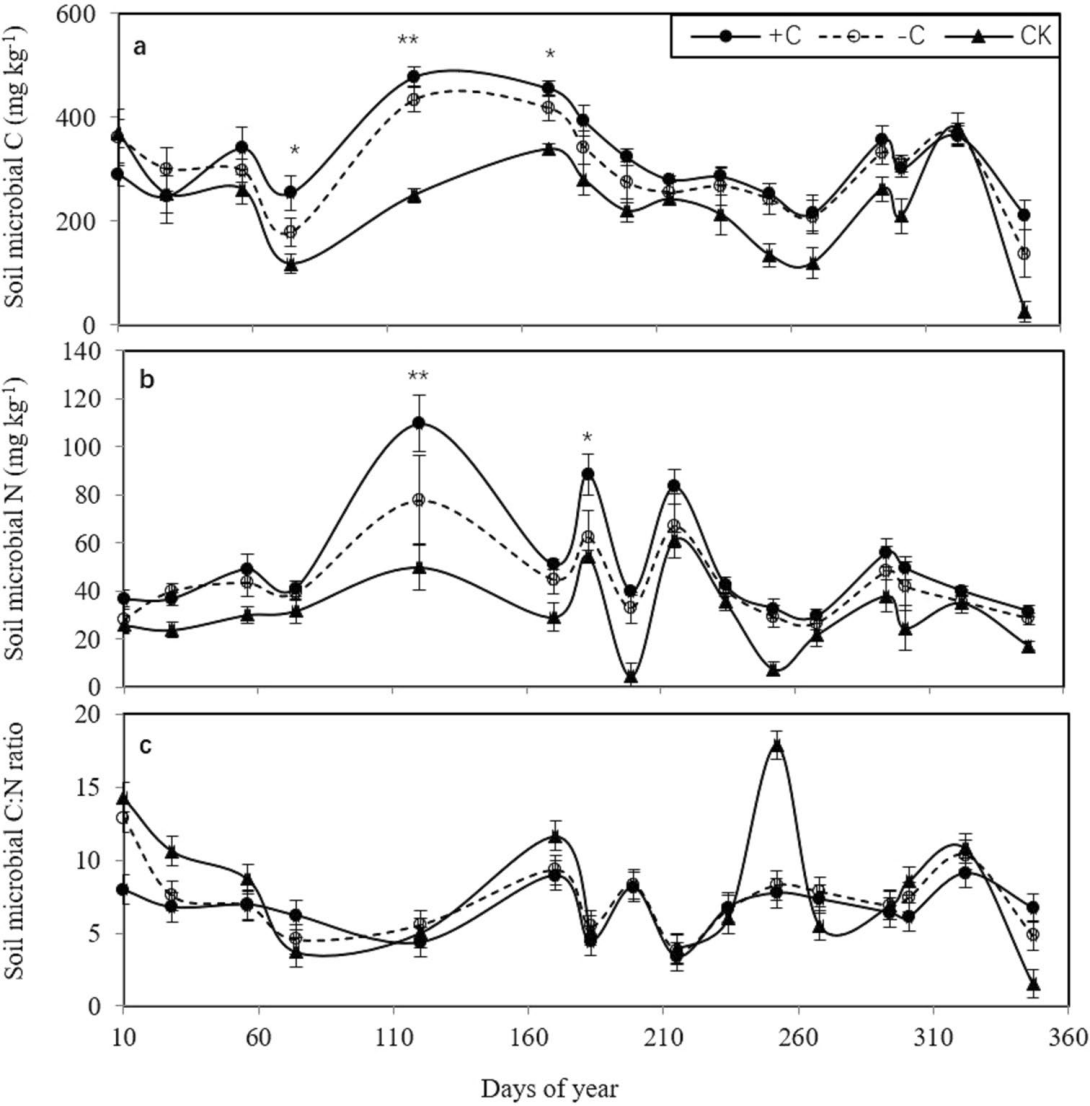
Variation in the soil microbial carbon, nitrogen and C:N ratio among the three treatments (cover crops with chicken grazing (+ C), cover crops without chicken grazing (− C), and bare fallow field (CK). *, ** indicate significant differences at the 0.05 and 0.01 levels, respectively.

The soil microbial carbon and nitrogen contents in the plot with cover crops were higher than those in the bare fallow field (CK, Fig. 1a,b). The peak of the soil microbial carbon and nitrogen contents occurred 120 days after chicken grazing, reaching 478.6 and 19.1 mg kg^−1^ in the + C treatment and 433.9 and 23.5 mg kg^−1^, in the − C treatment, respectively. There was a significant difference among the + C, − C, and CK treatments. In addition, the soil microbial C:N ratio in the cover crop plot was not higher than that in the CK plot, while in the late rice harvesting stage (347 days after chicken grazing), the soil microbial carbon and nitrogen in the + C and − C treatments were higher than that in the CK plot (Fig. 1c). The results suggest that in situ chicken manure input to the plots with cover crops and chickens increases the carbon and nitrogen sources available to the soil microorganisms, which may also stimulate cover crop growth and development, and further increase root exudates for the soil microorganisms.

### Dissolved organic carbon and nitrogen

**Figure 2:**
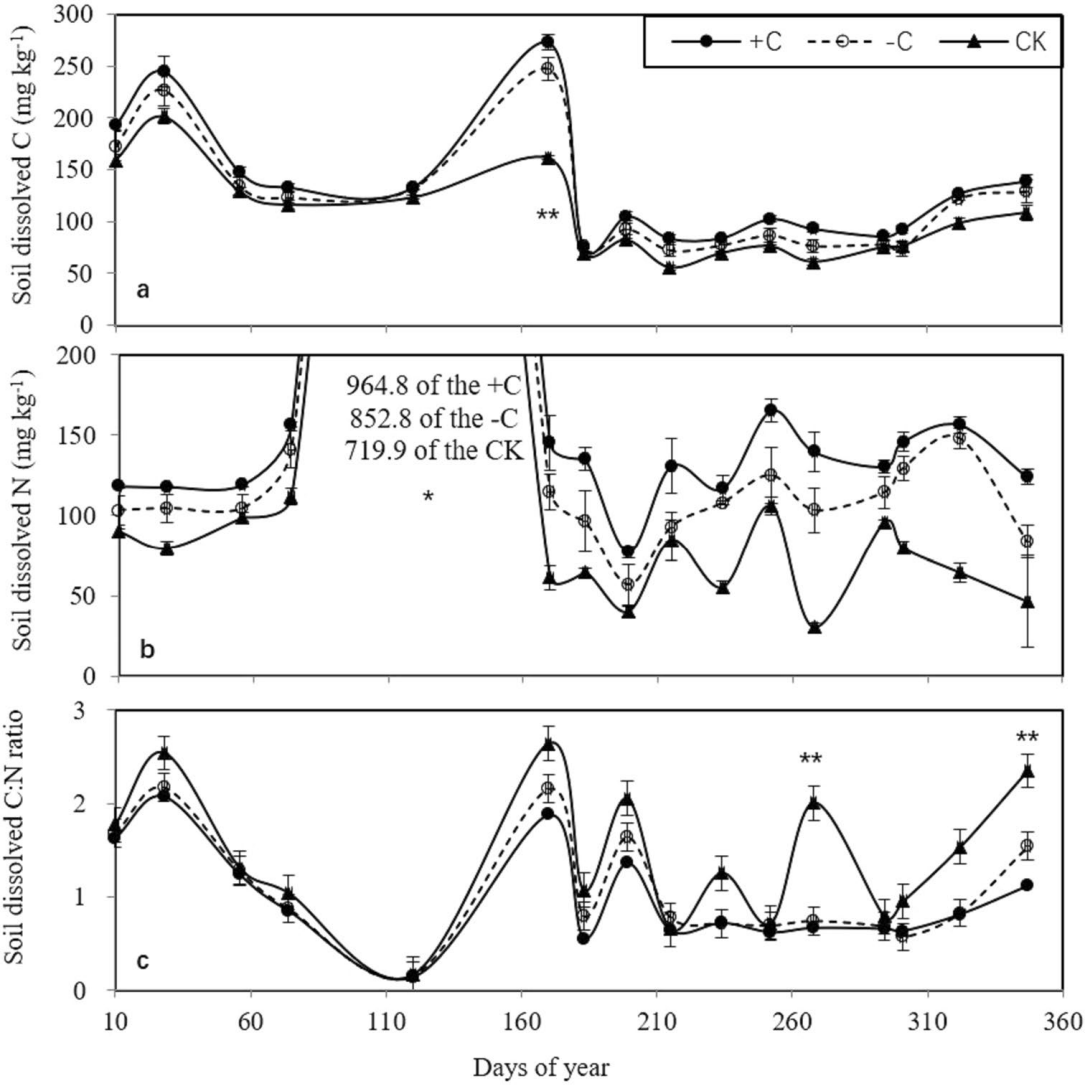
Variation in the soil dissolved nitrogen, carbon and C:N ratio among the three treatments (cover crops with chicken grazing (+ C), cover crops without chicken grazing (− C), and bare fallow field (CK). *, ** indicate significant differences at the 0.05 and 0.01 levels, respectively.

The dissolved organic carbon and nitrogen (DOC and DON, respectively) in the plots with cover crop were higher than those in the bare fallow field (CK, Fig. 2a,b). Similarly, the peak DOC was observed 170 days after chicken grazing, reaching 272.7 mg kg^−1^ for the + C treatment and 247.7 mg kg^−1^ for the − C treatment. The peak DON occurred 120 days after chicken grazing, reaching 964.8 mg kg^−1^ for the + C treatment and 852.8 mg kg^−1^ for the − C treatment. A significant difference was found among the + C, − C, and CK treatments. The dissolved organic C:N ratio in the cover crop plots was lower than that in the CK treatment, while the dissolved organic C:N ratio in the + C and − C treatments in the late rice harvesting stage (347 days after chicken grazing) was significantly lower than that in the CK (Fig. 1c).

### Soil ammonium and nitrate nitrogen

**Figure 3:**
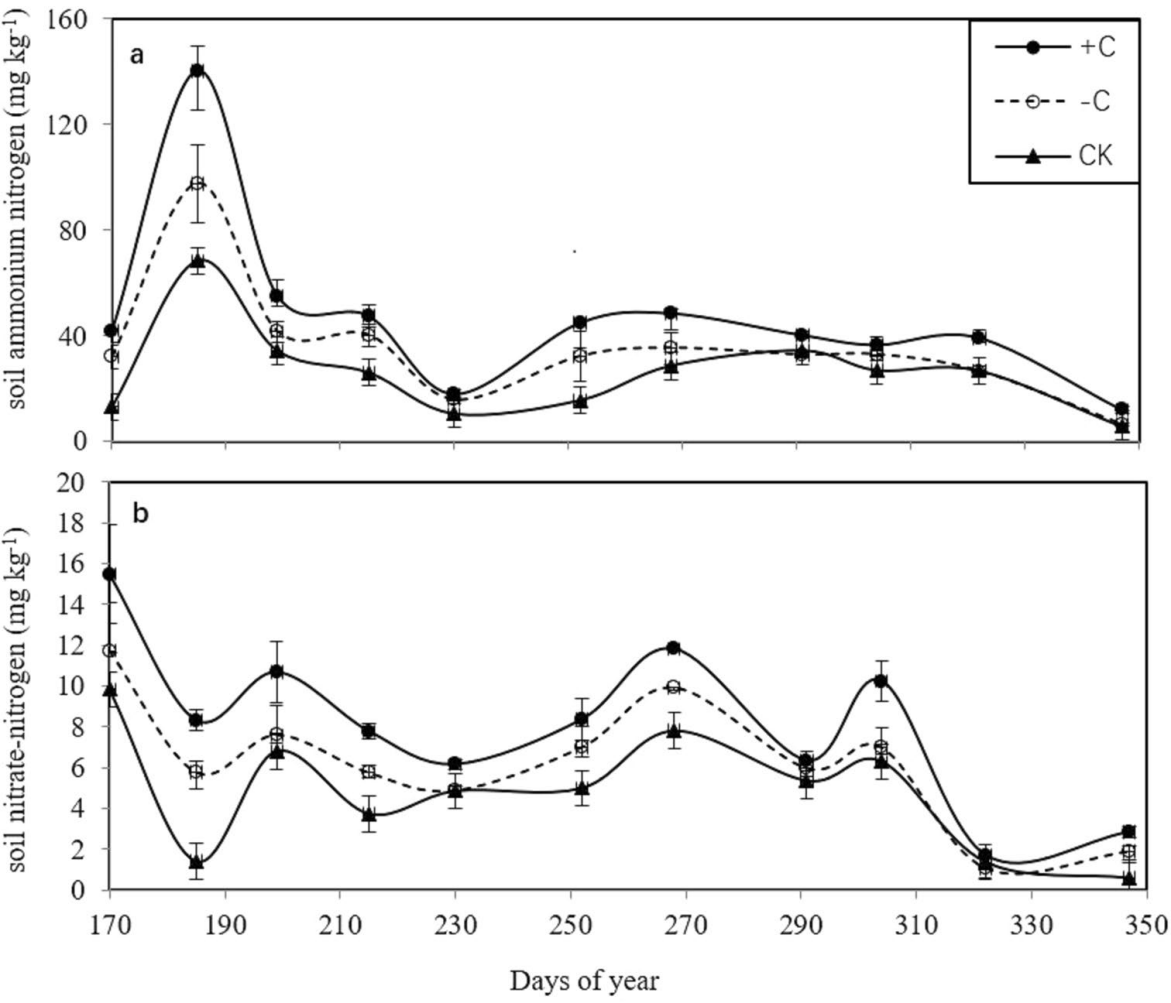
Variation in the soil ammonium nitrogen and nitrate-nitrogen among the three treatments (cover crops with chicken grazing (+ C), cover crops without chicken grazing (− C), and bare fallow field (CK).

Throughout the rice growth period, the soil ammonium and nitrate nitrogen in the plots with cover crops were higher than those in the bare fallow field (CK, Fig. 3a,b). The soil ammonium and nitrate nitrogen in the + C treatment were higher than those in the − C treatment.

### Daily and annual CH_4_ and CO_2_ net ecosystem exchange

**Figure 4:**
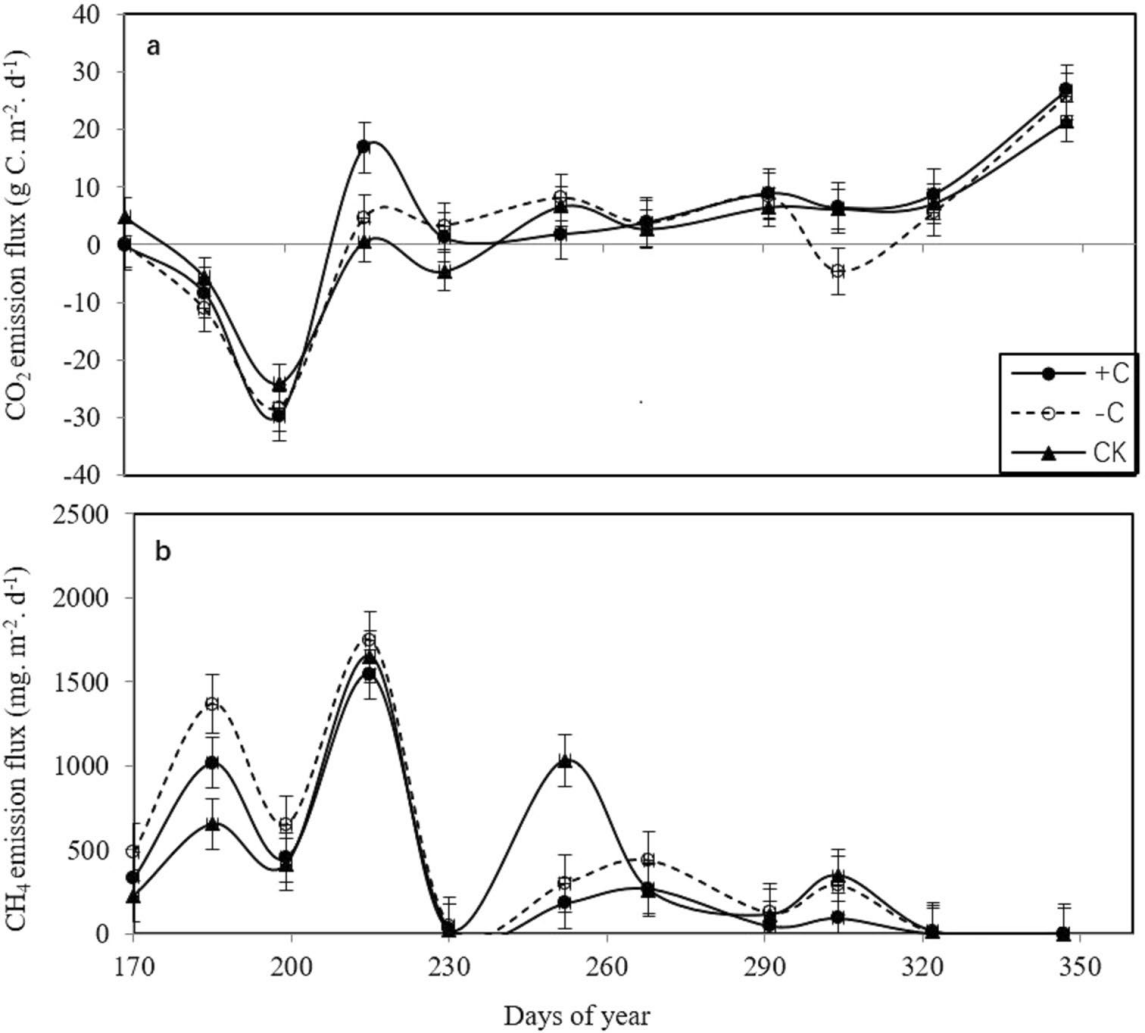
Variation in the CO_2_ and CH_4_ fluxes among the three treatments (cover crops with chicken grazing (+ C), cover crops without chicken grazing (− C), and bare fallow field (CK).

During the rice growth period, the peak CO_2_ and CH_4_ fluxes occurred during the early rice season (Fig. 4). The CH_4_ fluxes in the plots with cover crops were higher than those in the bare fallow field (CK, Fig. 4b), while the peak CH_4_ fluxes in the + C treatment were lower than those in the − C treatment. The annual CH_4_ emissions in the + C treatment were 806.5 kg ha^−1^ (Table 2), 10.6% lower than those in the CK treatment and 26.9% lower than those in the − C treatment. The annual CO_2_ emissions of the + C treatment were 8,214.0 kg ha^−1^, with a significant difference observed between the + C treatment and the other two treatments. The 100-year global warming potentials (GWPs) of the + C treatment were 6.2% lower than those of the − C treatment (Table 2). Although the GWPs in the plots with cover crops increased by 8.7% compared with those in the the CK treatment, there was not a significant difference between the plots with cover crops and the CK treatment.

**Table 2:** CO_2_ and CH_4_ emissions and their 100 a global warming potentials (GWPs) among the three treatments [cover crops with chicken grazing (+ C), cover crops with no chicken grazing (− C), and bare fallow field (CK)].

Treatment	CO_2_ emissions (kg ha^−1^)			CH_4_ emissions (kg ha^−1^)			GWPs (kg CO_2_-eq ha^−1^)
	Early season	Late season	Total	Early season	Late season	Total	
+ C	− 4,208.1b	12,142.2a	8,214.0a	681.3a	125.2b	864.3b	29,820.4a
− C	− 6,888.3a	9,227.2a	2,272.6c	857.5a	246.0a	1,180.4a	31,783.3a
CK	− 6,113.5a	10,331.8a	4,265.1b	608.6a	294.0a	963.0a	28,340.9a

Different lowercase letters in the same column among the different treatments indicate significant differences at the 0.05 level.

### GPP, R_eco_ and NEE

**Figure 5:**
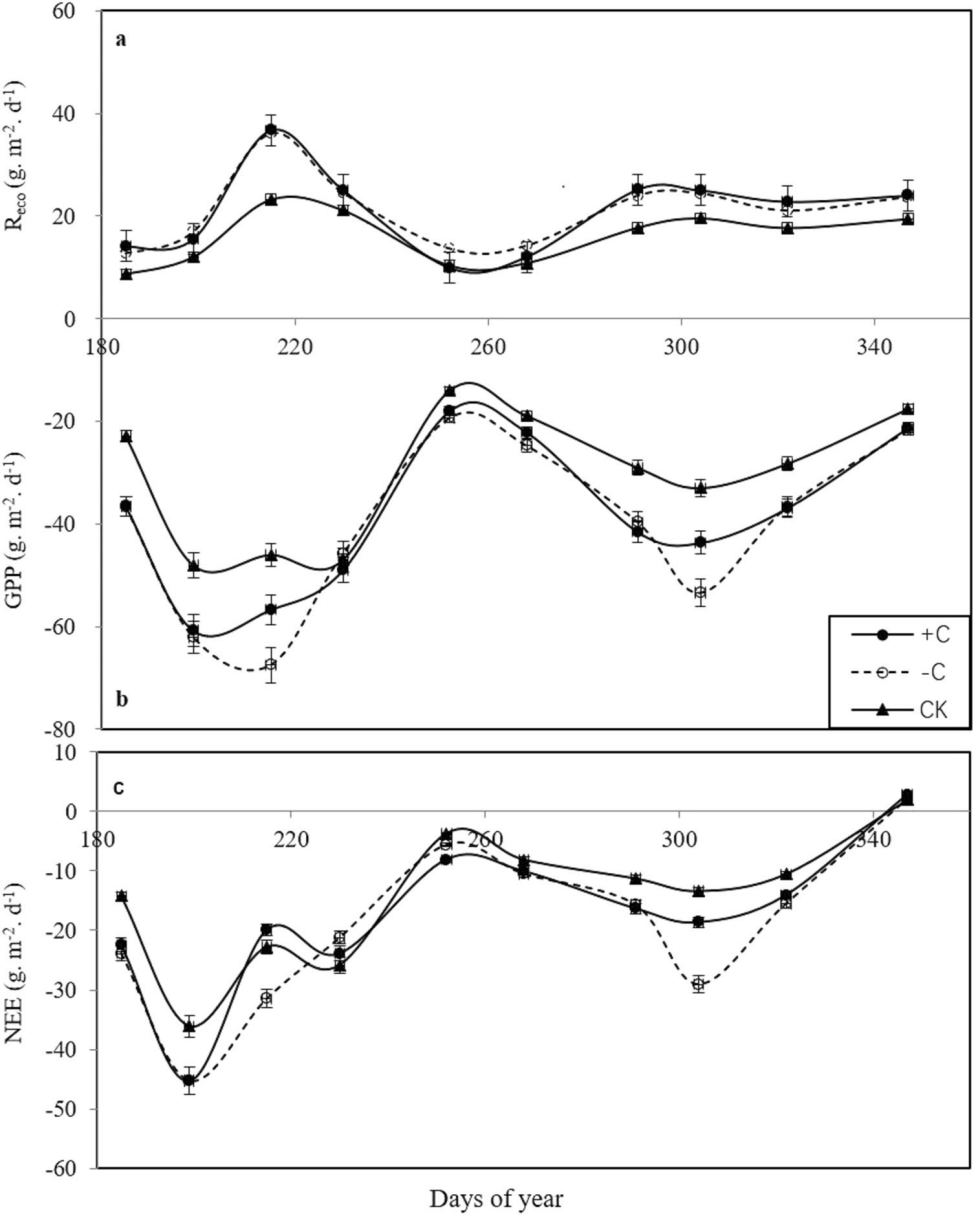
Variation in the gross primary production (GPP), ecosystem respiration (R_eco_), and net ecosystem exchanges (NEEs) among the three treatments (cover crops with chicken grazing (+ C), cover crops without chicken grazing (− C), and bare fallow field (CK).

In the rice growth period, the gross primary production (GPP) in the plots with cover crops and chicken grazing and the plots with cover crops and no chicken grazing was higher than that in the bare fallow field (CK, Fig. 5b). Moreover, the change in ecosystem respiration (R_eco_) was similar to the change in GPP (Fig. 5a). Except during the late rice harvesting season, the net ecosystem exchanges (NEEs) were shown as CO_2_ uptake (negative value, Fig. 5c).

### Yield

The rice yield in the plots with cover crops and chicken grazing increased by 12.3% compared with that in the CK treatment (Table 3). The yield of the + C treatment was 3.8% higher than that of the − C treatment, but the difference between the + C treatment and − C treatments was not significant. For the yield components, a higher number of panicles per unit area m^−2^ may be the main factor for increasing the rice yield in the plots with cover crops; a significant difference in panicles m^−2^ existed between the + C treatment and CK treatment.

**Table 3:** Yield and its components among the three treatments [chicken grazing (+ C), cover crops with no chicken grazing (− C), and bare fallow field (CK)].

Season	Treatment	Yield (t ha^−1^)	Panicle m^−2^	Spikelets per panicle	Filling ratio (%)	Grain weight (mg)
Early season	+ C	6.3a	306a	115a	76.1a	26.6a
	− C	6.1ab	282ab	118a	75.0a	26.8a
	CK	5.6b	245b	113a	79.8a	27.6a
Late season	+ C	7.1a	411a	87b	82.8a	27.5a
	− C	6.8a	364a	93a	79.9a	27.3a
	CK	6.1b	318b	93a	83.6a	27.4a

Different lowercase letters in the same column among the different treatments indicate significant differences at the 0.05 level.

## Discussion

The results of this study could be used to reduce inorganic nitrogen fertilization by approximately 33% compared with the level of inorganic nitrogen applied by Huang et al.^6^ in the same region. Grazing chickens in plots with cover crops (+ C) could improve soil carbon and nitrogen transformations and increase the double-season rice grain yield. The winter fallow period is a suitable window for renewing and improving soil fertility in the double-season rice cropping system in southern China. Our results suggest that the + C treatment is an effective measure for improving soil fertility and reducing nitrogen application. It has been recommended to use the cover crops (green manure) planted in winter fallow fields in rice paddy soil^5,7^; however, our data indicated that the total carbon, nitrogen, and labile carbon and nitrogen contents in the + C treatment were higher than those in the treatment with cover crops alone (− C), and the yield of the + C treatment was 3.8% higher than that of the − C treatment. Therefore, the combination of chicken grazing and cover crops in the winter fallow field is a win–win model for improving soil fertility and increasing rice grain yield while reducing the quantity of inorganic nitrogen application. Moreover, using symbiotic farming technologies, such as cover crops and chicken farming, in fallow winter fields promotes green manure applications, because chicken grazing could increase the economic benefits for organic farming.

Organic chicken manure^16,17^ and green manure application significantly increased the contents of labile C and N measured by dissolved organic carbon (DOC) and dissolved organic nitrogen (DON). DOC and DON affect the supply and effectiveness of nutrients^18,19^. For example, Jones et al.^17^ reported that DON constitutes a major soluble N pool in most soils and that plant roots have the potential to access some of this pool. Magill and Aber^15^ indicated that labile carbon appears to increase net N immobilization rather than net N mineralization. In this study, the DOC in the cover crop treatments in the winter fallow field was higher than that in the CK treatment, and a similar trend was found for the DON. Further, the DOC and DON in the + C treatment were higher than those in the − C treatment. Our data suggested that the use of a combination of chicken grazing and cover crops in winter fallow fields could improve the soil nutrient potential and the effectiveness of the nutrients and increase the amount of soil nitrogen available for direct and indirect absorption by plants. Evidence of higher double-season rice yields in the + C treatment indicated that the use of chicken grazing and cover crops could provide more DON and DOC to the plants.

Fresh manure application and crop straw recycling can cause a significant stimulation of CH_4_ emissions, as these materials provide abundant and fresh C sources for CH_4_ production in aerobic rice fields^8,9^. Our results showed that the CH_4_ emissions in the − C treatment were higher than those in the CK treatment. Taking CH_4_ and CO_2_ emissions and crop yields into account, previous studies have suggested that decomposed organic matter, such as biogas residue, can improve long-term soil fertility without an increase in yield-scaled GWPs^20,21^. Our results showed that the + C treatment had no significant stimulatory effect on the total CH_4_ emissions, especially in the late rice season. Different exogenous carbon inputs (e.g., fresh manure and/or green manure) could significantly change the abundance and community composition of soil methanogens and methanotrophs. Wang et al.^22^ found that the microbial mechanism associated with the reduction in CH_4_ emissions in fields amended by biochar was the variation in the soil methanogen to methanotroph abundance (*mcrA/pmoA* ratio), which is mainly ascribed to increased soil ${\text{NH}}_{4}^{ + }$, DOC content, and porosity. Soil DOC could enhance methanotroph growth directly^23,24^. The dynamic determination of soil nutrients in this study also showed that the DOC and soil ammonium in the + C treatment were higher than those in the CK treatment. Therefore, the + C treatment reduced CH_4_ emissions, probably by providing more labile organic C compounds and even increasing the abundance of soil methanotrophs.

However, CH_4_ emissions in the early rice season were significantly higher than those in the late rice season, which was in contrast with the report of Feng et al.^21^. A possible reason was that the exogenous carbon input was concentrated in the early season, and the early rice straw was removed in the late season. In this study, the exogenous carbon input in the early season included rice straw from the previous late season, green manure and chicken manure, and the extra ${\text{NH}}_{4}^{ + }$–N and DOC from the exogenous carbon input were exhausted during the early season. The soil Eh and DOC contents were key determinants of the soil CH_4_ emissions^14,25,26^. On the other hand, the DOC and soil microbial carbon in the early season were higher than those in the late season, and the peak values of DOC and soil microbial carbon were found in the early season, therefore providing higher C sources for CH_4_ production in the early season than in the late season. This phenomenon suggested that the early rice season was more suitable than the late rice season for exogenous carbon input. The average air temperature from the transplantation stage to the full-heading stage was 22.8 °C in the early season and 26.7 °C in the late season. The soil temperature increased with the average air temperature, although there was not a linear relationship between these variables, and soil temperature increase can cause a significant stimulation of CH_4_ emissions from rice fields^27^.

## Conclusions

Cover crop-chicken farming in a winter fallow field was an effective model to reduce the amount of carbon emissions and fertilization and significantly improve soil nutrients and rice yields. It provides an alternative technological approach to improve soil fertility under intensified rice cultivation.

## Materials and methods

### Experimental site and test cultivars

A field experiment of cover crop planting in a winter fallow field was conducted in Changsha (28° 11′ N, 113° 04′ E), Hunan Province, China, from 2014–2015. The soil in the experimental field was tidal clay, with 1.16% organic carbon, 0.17% total N, and a pH of 6.15.

### Experimental design and field management

A randomized block experiment was established with 3 different treatments, including cover crops (*Lolium* spp. and *Astragalus sinicus*) with chicken grazing (+ C), cover crops without chicken grazing (− C), and a bare, fallow field (CK). Each field plot covers 140 m^2^, and there were three replications. To prevent the movement of water between adjacent plots, ridges were covered with a plastic sheet inserted into the soil to a depth of 0.5 m.

Ryegrass and milk vetch were planted on October 10th, 2014, at seed densities of 23 and 40 kg ha^−1^, respectively. Thirty-day-old yellow chickens were introduced into the field on November 25th. To ensure the homogeneity of the chicken manure inputs, a 3 m × 3 m cage was used during the process of chicken grazing. There were 30 chickens in each cage. Five kilograms of corn flour was fed to the chickens in each cage daily. The corn flour was 1.8% nitrogen. The cage was moved every 7 days in the chicken-grass plot until February 2, 2015. The quantity of in situ chicken manure input into the system within the symbiotic period (69 days) in these plots was estimated to be 96.3 t ha^−1^ by collecting the chicken waste in an underground container. The underground container was a square with a side length of 50 cm and a height of 10 cm. There were 3 symbiotic periods in these plots, and the chicken waste samples were collected every 12 h for three days. On March 27th, 2015, the average aboveground biomass of the cover crops was 11.7 t ha^−1^ in the + C plot and 14.4 t ha^−1^ in the − C plot. All the procedures used in this experiment were conducted in accordance with the Chinese Guidelines for Animal Welfare. The experimental procedures performed in the current study were approved by the Hunan Agricultural University Institutional Animal Care and Use Committee (Changsha, China). Furthermore, all the experimental protocols, including animal handling, were performed humanly, and animal welfare was specially considered. We further confirmed that no animals were harmed or stressed during the experimental period.

The cover crops were incorporated into the soil on March 27th, and all the plots were used to grow double-season rice. The early rice cultivar ‘Zhongjiazao 17’ and the late rice cultivar ‘Xiangwanxian 12’ were used in the experiment, and their growth durations were 109 days and 115 days, respectively. Rice seedlings were transplanted on May 5th and harvested on July 12th for the early-season rice, followed by the late-season rice, which was transplanted on July 25th and harvested on October 30th. The seedlings were 35 and 25 days old in the early and late seasons, respectively. The transplantation density was 30 hills m^−2^ for the early rice season and 25 hills m^−2^ for the late rice season.

We supplied nitrogen (N) in the form of urea, calcium superphosphate for phosphorus pentoxide (P_2_O_5_), and potassium chloride for potassium oxide (K_2_O) in the rice growing season. The quantity of N supplied was 74 kg ha^−1^ in the early rice season and 102 kg ha^−1^ in the late rice season. Urea was applied three times during the rice season; the ratio of tillering fertilizer to panicle fertilizer (grain fertilizer) was 70:30 in the early rice season and 50:50 in the late rice season. The quantity of P_2_O_5_ and K_2_O supplied was 60 kg ha^−1^, and the same quantity was applied in both seasons. Potassium chloride was applied twice during the rice season, 50% as basal fertilizer and 50% as tillering fertilizer. The calcium superphosphate was applied as a basal fertilizer before transplantation. Water management was performed according to the technology used for double rice cropping systems (local high-yield cultivation) (Table 4).

**Table 4:** Experimental design^16^.

Season	Cover crops with chicken grazing (+ C)		Cover crops without chicken grazing (− C)	Bare fallow field (CK)
Winter	No fertilization	Oct. 15, 2014: Cover crop seeding	Oct. 15, 2014: Cover crop seeding	Oct. 15, 2014: No cover crop
		Nov. 25, 2014–Feb. 2, 2015: Chicken grazing		
		Mar. 27, 2015: Tillage	Mar. 27, 2015: Tillage	Mar. 27, 2015: Tillage
Early rice	Fertilization	The amount of fertilizer input was reduced by 30%, and nitrogen was not included in the base fertilizer May 4, 2015: base fertilizer: P_2_O_5_ 60 kg·ha^−1^; K_2_O 30 kg·ha^−1^; May 17, 2015: tiller fertilizer: N 51.8 kg·ha^−1^; Jun. 16, 2015: panicle fertilizer: N 22.2 kg·ha^−1^; K2O 30 kg·ha^−1^		
Late rice	Fertilization	The amount of fertilizer input was reduced by 10%, and nitrogen was not included in the base fertilizer Jul. 24, 2015: base fertilizer: P_2_O_5_ 60 kg·ha^−1^; K2O 30 kg·ha^−1^; Aug. 3, 2015: tiller fertilizer: N 51.0 kg·ha^−1^; Aug. 30, 2015: panicle fertilizer: N 30.6 kg·ha^−1^; K_2_O 30 kg·ha^−1^; Sep. 12, 2015: grain fertilizer: N 20.4 kg·ha^−1^		

### Soil chemical properties

Soil samples from the 0–20 cm soil layer were used to determine the soil chemical properties. The samples were collected during cover crop harvesting, early rice harvesting and late rice harvesting. The soil samples were air dried and the soil organic matter was determined using K_2_Cr_2_O_7_ and concentrated H_2_SO_4_ and heating. The soil total N was determined with the Kjeldahl method, which involved two steps: (1) the digestion of the samples to convert organic N into ${\text{NH}}_{4}^{ + }$–N and (2) the determination of ${\text{NH}}_{4}^{ + }$–N in the digest. The soil C:N ratio was calculated by dividing the SOC concentration by the TN concentration. Soil ammonium N was analyzed using indophenol blue colorimetry. Soil nitrate–N was analyzed using ultraviolet spectrophotometry.

### In situ CH_4_ and CO_2_ flux measurements

During the rice growing season, in situ CH_4_ and CO_2_ flux were measured with a static chamber by circulating the gas within the chamber and pipes of an ultraportable greenhouse gas analyzer (CH_4_/CO_2_/H_2_O Analyzer; Los Gatos Research Corp., USA). The static chamber was a square with a side length of 50 cm and a height of 120 cm. A fluted base consistent with the static chamber was inserted in the soil in advance. On the sampling dates, daytime samples were collected from 9:00–11:00 a.m. and 15:00–17:00 p.m., and nighttime samples were collected from 19:00–21.00 p.m. The testing time in each plot was 5 min. The sampling dates were 170, 185, 199, 215, 230, 252, 268, 291, 304, 322, and 347 days after the chickens were introduced into the field. The samples were collected at intervals of 14 days, plus or minus one day if the weather forecast for a sampling date was rainy.

The temperature inside the static chamber needs to be accurately recorded at a soil depth of 3 cm. Plants (excluding the border plants) were sampled from a 0.24 m^2^ area of each plot on the sampling date. The plant samples were manually separated into leaf and straw and/or grains. The volume of the plant samples was measured with drainage. The effective volume in the chamber was reduced to subtract the internal plant volume from the chamber. The leaf area was determined with a leaf area meter (LI-3000A, LICOR, Lincoln, NE, USA). Lastly, the plant samples were oven-dried at 70 °C to constant weight to determine the aboveground biomass.

The CO_2_ (F, g m^−2^ day^−1^) and CH_4_ (F, mg m^−2^ day^−1^) fluxes were calculated using the following formula (Eq. 1):

1 $$ {\text{F}} = \frac{{{\text{P}} \times {\text{V}}}}{{{\text{R}} \times {\text{A}} \times \left( {{\text{T}} + 273.15} \right)}} \times \frac{{{\text{dc}}}}{{{\text{dt}}}}, $$

where P is the atmospheric pressure under standard conditions (101.2237 × 10^3^ Pa); V is the effective volume in the chamber (m^3^), the difference between the volume of the static chamber and the volume of the plant, fan and temperature recorder; R is a gas constant (8.3144 J⋅mol^−1^ K^−1^); A is the area of the chamber cover (m^2^); T is the average temperature at testing time inside the chamber (°C); and dc/dt is the rate of change in the concentration of CO_2_ and CH_4_.

To accurately calculate the CO_2_ and CH_4_ fluxes in the paddy field, the daytime and nighttime CO_2_ and CH_4_ fluxes on the sampling dates were calculated using the following formulas (Eq. 2–4):

2 $$ {\text{F}}_{{{\text{daytime}}}} = {\text{ S}}_{{{\text{daytime}}}} \times {\text{M}} \times \left( {{\text{F}}_{{1}} + {\text{F}}_{{2}} } \right)/{2,} $$

3 $$ {\text{F}}_{{{\text{night}}}} = {\text{ F}}_{{3}} \times {\text{S}}_{{{\text{night}}}} \times {\text{M,}} $$

4 $$ {\text{F}}_{{{\text{day}}}} = {\text{ F}}_{{{\text{daytime}}}} + {\text{F}}_{{{\text{night}}}} , $$

where F_1_, F_2_ and F_3_ represent the values at 9:00–11:00 a.m. and 15:00–17:00 p.m. on sunny days and 19:00–21:00 p.m., respectively; S is the day length (s day^−1^) on the sampling date; and M is the relative molecular mass of CO_2_ or CH_4_ (g mol^−1^).

Seasonal emissions in CO_2_ and CH_4_ were calculated using the following formula (Eq. 5):

5 $$ {\text{T }} = {\text{a}} \times {1}0 \times \left( {\mathop \sum \limits_{{{\text{i}} = 1}}^{{\text{n}}} [\frac{{{\text{F}}_{{\text{i}}} + {\text{F}}_{{{\text{i}} + 1}} }}{2}\left( {{\text{t}}_{{{\text{i}} + 1}} - {\text{t}}_{{\text{i}}} } \right)] + \frac{{{\text{F}}_{{\text{i}}} + {\text{F}}_{{\text{n}}} }}{2}} \right), $$

where T (g m^−2^) is the total seasonal emissions, F_i_ and F_i+1_ are the measured fluxes on two consecutive sampling days, t_i+1_ − t_i_ is the number of days between the two sampling dates, 10 is the conversion coefficient from g m^−2^ to kg ha^−1^, and a is the conversion coefficient of the rice growth period (86/61 in the early season and 132/96 in the late season).

In addition, the period from early rice harvesting to late rice transplanting is 13 days. The emissions were calculated using the following formula (Eq. 6):

6 $$ {\text{T}}_{{{\text{ER}} - {\text{LR}}}} = {\text{ T}}_{{{\text{ER}}}} /{86} \times {6}.{5} + {\text{T}}_{{{\text{LR}}}} /{132} \times {6}.{5,} $$

where T_ER-LR_ (g m^−2^) is the total emissions from early rice harvesting to late rice transplanting, T_ER_ and T_LR_ are the total seasonal emissions in the early rice season and late rice season, respectively, and 86 and 132 are the number of days from sowing to harvesting in the early rice season and late rice season, respectively.

### Soil microbe and dissolved carbon and nitrogen measurements

In 2014, soil was sampled from the 0–20 cm soil layer, and the sampling dates were 10, 28, 56, 74, 120, 170, 183, 199, 215, 234, 252, 268, 294, 301, 322, and 347 days after chicken grazing. Fresh soil samples were taken to determine the soil microbial carbon and nitrogen contents by chloroform fumigation-incubation and K_2_SO_4_ extraction. Soil microbial carbon (SMC, mg kg^−1^) = EC/0.38 and soil microbial nitrogen (SMN, mg kg^−1^) = EN × 0.45, where 0.33 and 0.45 are the conversion coefficients of SMC and SMN, respectively. EC and EN are the differences in organic carbon and nitrogen between fumigation and nonfumigation based K_2_SO_4_ extraction. In addition, other fresh soil samples were used to determine the soil dissolved carbon and nitrogen by K_2_SO_4_ extraction.

### Yield and its components

When the rice was mature, 10 hills were sampled randomly from a 5 m^2^ harvest area to determine the yield components. Panicle number was counted on each hill to determine the panicle number per m^2^. The panicles were hand-threshed, and the filled spikelets were separated from the unfilled spikelets by submerging them in tap water. Three subsamples of 30 g of filled spikelets and 3 g of unfilled spikelets were taken to count the number of spikelets. Based on the spikelets per panicle, the grain-filling percentage (100 × filled spikelet number/total spikelet number) was determined. The grain yield was determined from a 5 m^2^ area in each plot and adjusted to the standard moisture content of 0.14 g H_2_O g^−1^.

### Data analysis

The global warming potential (GWP) was the overall GWP of CH_4_ and N_2_O emissions per unit rice field (ha). The 100-year radiative forcing potential coefficients relative to CO_2_ were 25 and 298 for CH_4_ and N_2_O, respectively (IPCC, 2007). The net ecosystem exchange (NEE) was the value of F_daytime_, ecosystem respiration (R_eco_) was the value of F_nighttime_, and gross primary production (GPP) was the sum of the NEE and R_eco_. The means of the indexes were organized in Excel 2016. The SD (standard deviation) of the indexes were determined by descriptive statistics with a 95% confidence interval. Analysis of variance (ANOVA) and multiple comparisons were performed using Statistix ver. 8.0 (2004) to evaluate the effects of planting cover crops and chicken grazing on the SOC, STN, C:N ratio, DOC, DON, SMN, SMC, and grain yield and its components.
